# An assessment of existing surge capacity of tertiary healthcare system of Khyber Pakhtunkhwa Province of Pakistan using workload indicators for staffing need method

**DOI:** 10.1186/s12960-021-00663-3

**Published:** 2022-01-28

**Authors:** Muhammad Zeeshan Haroon, Inayat Hussain Thaver

**Affiliations:** 1 Department of Community Medicine, Ayub Medical College, Abbottabad, Pakistan; 2 Department of Community Health Sciences, Bahria University Medical & Dental College, Karachi, Pakistan

**Keywords:** Surge capacity, WISN, Healthcare system, Disasters, Emergencies

## Abstract

**Background:**

Globally the occurrence of disasters has increased more than fourfold during the last three decades. The main concern for the healthcare system responding to a disaster is its ability to deal with the sudden influx of patients and maintaining a certain level of surge capacity. Health workers are considered to be the major driving force behind any health system. Their role gets even more prominent during disasters or public health emergencies. With the lack of information on the health workforce in the tertiary healthcare system of Khyber Pakhtunkhwa, where most of the disaster surge is diverted, it is difficult to plan and respond to accommodate the sudden surge of patients.

**Methods:**

This was a mixed method cross-sectional survey conducted in all the tertiary care hospitals of Khyber Pakhtunkhwa province of Pakistan to assess the current staffing situation and surge capacity based on the current workload. Annual service statistics of 2018 were collected from all the tertiary care hospitals of the province. WISN was piloted with only one healthcare staff category, i.e., for doctors in Ayub Teaching Hospital before assessment in all the tertiary care hospitals was undertaken.

**Results:**

Overall, there were 1215 surplus doctors in medical and allied specialties and 861 doctors in surgical and allied specialties in the tertiary healthcare system. The health care system has an acute shortage of 565 emergency department doctors. The tertiary healthcare system of KP has an overall shortage of 1099 nurses. Based on the WISN generated numbers for doctors, the tertiary care system of KP has a combined healthcare staff (doctors and nurses) that can manage an additional surge of 6.3% of patients with the current patient workload.

**Conclusion:**

The tertiary health care system of the Khyber Pakhtunkhwa Province of Pakistan does not possess the required ≥ 20% HR surge capacity indicating that the tertiary healthcare system is poorly prepared for disasters or public health emergencies. The lack of nursing staff, more than the doctors, is the major reason behind the lack of HR surge capacity of the tertiary health care system.

## Background

Disasters are catastrophic events that cause widespread death and misery. Globally the occurrence of disasters has increased from 120 per year in the 1980s to around 500 in the twenty-first century [1]. They have far-reaching social and economic effects on communities. They jeopardize the health of local communities in terms of the number of dead or injured and disrupt local health services due to loss of health staff, structural damage to healthcare facilities, and overburdening of healthcare services [2]. A healthcare system can easily be overwhelmed during disasters by a large and sudden influx of individuals in need of immediate healthcare.

Pakistan is one of the few countries in the world that are extensively affected by both natural and man-made disasters [3]. In the last two decades, Pakistan has seen an unprecedented number of disasters whether natural or manmade. During this period, the disasters have cost Pakistan around US$126.79 billion in direct and indirect costs [4]. The province of KP has especially been affected by the man-made disaster in the form of suicide attacks, mass shootings, and armed conflicts in addition to natural disasters. Pakistan was at one point, ranked third after Iraq and Afghanistan in terms of suicide bombings [5]. Till June 2018, a total of 479 incidents of suicide bombings occurred resulting in 7291 deaths and more than 15,400 injuries [6]. Most of the burden of these conflicts has been borne by the tertiary healthcare facilities of Khyber Pakhtunkhwa. The main concern for the healthcare system and hospitals responding to a disaster is their ability to deal with the sudden and rapid influx of disaster victims or patients and maintaining a certain level of preparation for disaster that improves surge capacity.

Surge capacity is an essential component of all the elements covering disaster preparedness. It represents the ability of the healthcare system to handle an explosive patient inflow. There is no standardized or uniform definition of surge capacity. However, broadly surge capacity can be described as an ability of a healthcare system to scale up swiftly beyond its regular norms to provide for the increased patient load during disasters or public health emergencies [7].

Although, there is no consensus on the single definition of surge capacity, yet literature shows a considerable consensus on the elements of surge capacity. During the evolution of this concept, bed availability was considered as the sole indicator of surge capacity [8, 9]. However, as the concept evolved, academics became critical of relying solely on the bed numbers as an indicator of surge capacity and identified Staff, Stuff, System, and Structure as the components determining surge capacity [10–12]. Studies have identified that instead of bed occupancy, the availability of the staffed beds can be the major limiting factor of the surge capacity in accommodating and providing treatment patients during disasters. Several surge need assessment studies have shown that hospitals must have the ability to augment 20% of the staffed beds to ensure meeting the health needs of disaster victims [13–15]. These studies highlight the need and importance of adequate human resources in addition to physical logistics for effective disaster response by the health system.

The health workforce is considered to be the major driving force behind any health system. Their role gets even more prominent and important during disasters or public health emergencies that require surge capacity by building the resilience of the health systems [2]. Despite the importance of human resources in the effective functioning of health systems, there is a significant shortage of appropriate health workers. It is estimated that due to a shortage of 17.4 million health workers globally, between 1 and 3 billion people have no access to health care services [2, 16, 17]. Pakistan is one of the 57 countries identified by WHO that are facing a health workforce crisis [18]. The HRH situation in Khyber Pakhtunkhwa (KP) province is no different from the overall country’s HRH situation. According to a report published by Govt. of the KP, only 56.6% sanctioned posts of doctors and 68.1% sanctioned posts of nurses are filled in primary and secondary health care system [19]. No HRH data are available for the tertiary healthcare system of the province.

Several tools have been developed by WHO and partners to assess the HR needs in health systems [20–22]. Most of these tools employ simple approaches like using health workforce-to-bed or population ratio to generate the required numbers. However, for accurate estimation of HR surge capacity, the mere availability of the number of healthcare providers (HCPs) will not suffice. To ensure and prepare for an HR surge capacity of ≥ 20%, hospitals and health systems must take into consideration both numbers and workload of HR. Workload Indicators of Staffing Needs (WISN) which estimates staffing needs based on the actual workload of the healthcare staff may prove to be an ideal tool to assess the existing HR surge capacity of the tertiary healthcare system and its constituent hospitals. This research aims to estimates the existing HR surge capacity of the tertiary healthcare system of Khyber Pakhtunkhwa using WISN by taking into account both existing numbers and staffing needs based on actual workload. Accurate estimation of HR surge capacity will enable the tertiary healthcare system and its constituent hospitals to plan, allocate, and re-appropriate resources to manage victims during disasters and public health emergencies.

## Methods

This was a mixed method cross-sectional survey conducted in all the tertiary care hospitals of KP province of Pakistan namely Khyber Teaching Hospital (KTH), Lady Reading Hospital (LRH), Ayub Teaching Hospital (ATH), Hayatabad Medical Complex (HMC), Medical Teaching Institute Dera Ismail Khan (MTI DI Khan), Mardan Medical Complex (MMC), Medical Teaching Institute Bannu (MTI Bannu) and Saidu Group of Teaching Hospitals (SGTH) to assess the current staffing needs and situation based on the workload of the patients and HR surge capacity. This study was conducted between March and October of 2019. The methodology to conduct this assessment was explicitly based on the directions provided in the WHO’s “Workload Indicators of Staffing Need; User’s Manual” [23]. Ethical approvals were obtained from the relevant ethical review committees and data collection permission was obtained from the Hospital Directors/Medical Directors of the surveyed hospitals.

As per the WISN guidelines, two committees, Technical Task Force and Expert Working Group were formed. Technical Task Force was responsible for the implementation of WISN in KP. This team comprised WISN country resource person, national and internationally trained experts of WISN, health systems experts, and statistician. The Expert Working Group was constituted to define the main workload components and subsequent determination of activity standards for the identified workload components. The expert working group consisted of a wide range of experts from nurses and doctors belonging to every clinical specialty present in the teaching hospitals of Khyber Pakhtunkhwa. The following steps were carried out in chronological order:

1. Determination of priority cadres.
2. Estimation of available working time.
3. Defining workload components.
4. Setting activity standards.
5. Establishing standard workloads.
6. Calculating allowance factors.
7. Determination of staff requirements based on WISN.
8. Analysis and interpretation of WISN results.

It was decided to choose doctors and nurses for estimation of staffing need and surge capacity after consulting with the technical task force. The rationale for choosing doctors and nurses for this assessment is related to their role in managing the surge of patients in tertiary care hospitals during disasters. Disaster literature highlights the importance of doctors and nurses during disaster response and designates them as an essential component in healthcare delivery during routine and disaster scenarios [24–29]. Additionally, the workload components for doctors and nurses are well defined in the tertiary care system of KP.

The HCPs of KP are entitled to 25 casual leaves as per the Govt. of Khyber Pakhtunkhwa Leave rules [30]. Under the same rules, they are also entitled to other leaves which include earned leave, ex-Pakistan leave, maternity leave, recreation leave, leave prior to retirement, extraordinary leave, medical leave, special leave, quarantine leave, study leave, and leave for Hajj/Umrah. The HCPs also avail national holidays in addition to above mentioned categories of leave and in case they are on duty on the designated holidays, they are compensated with time off on a regular day.

Personal leave records were obtained from the HR department and duty registers of the eight tertiary care hospitals. Confidentiality was ensured by only obtaining leave records by designation and categories of HCPs. Total leaves under all the categories were calculated for both doctors and nurses which were then divided by the number of staff of each cadre in that hospital to get an average leaves per cadre per year. Average annual leaves for doctors and nurses were calculated to be 55 and 50 leaves, respectively.

Both doctors and nurses are required to work 6 days a week in all the tertiary care hospitals except LRH, where the nurses are working 5 days a week. The working hours for doctors are 36 h a week and for nurses, it is 48 h a week except for LRH, where nurses are required to work 40 h a week. Based on the possible available days and working hours/day, the available working time (AWT) for doctors was calculated as 1542 h in a year; whereas, the AWT for nurses was calculated as 2096 for all the tertiary care hospitals except LRH, where AWT for nurses was 1680 annual hours.

The workload components were defined by inviting the expert working groups from both nurses and doctors for focus group discussions (FGDs). On the advice of the technical task force, the expert working groups of doctors and nurses were further subdivided into the expert groups of medical and allied specialties (General Medicine, Pulmonology, Gastroenterology, Oncology, Cardiology, Pediatrics, Neurology, Psychiatry, Dermatology, etc.), surgical and allied specialties (General Surgery, Neuro Surgery, Orthopedics, Otolaryngology, Ophthalmology, Thoracic Surgery, Gynecology, Urology, etc.), and emergency department (ED) expert working group. This decision was taken based on the assumption that the workload components of surgical specialties, medical specialties, and ED differ from each other, and workload components can be grouped for similar specialties.

Twenty-four focus group discussions were carried out in four locations across the province. Six FGDs were arranged at each site. One FGD was arranged for professorial staff/consultant doctors related to medical and allied specialties and the emergency department. Another FGD was arranged for training medical officers (residents) and medical officers of medical and allied specialties and ED. Similar FGDs were arranged for surgical and allied specialties. For nurses, FGD was arranged for nurses working in medical and allied and ED specialties and another for nurses working in surgical and allied specialties.

The participants for FGDs were selected using non-probability purposive sampling. They were selected based on their knowledge and expertise in the related field. Before the start of the FGDs, based on the WISN user manual, an interview guide was made which included 12 discussion points related to workload indicators. The FGDs were carried out by the researcher and the two trained assistant moderators. Participants were briefed on the WISN in detail and the objectives of FGD were highlighted. The participants were asked to free list the activities they thought a well-motivated and qualified health care worker belonging to their specialty should perform at a locally acceptable professional standard of care. Participants were asked to identify Support Activities, Health Service Activities, and Additional Activities. For additional activities, participants were asked to identify workload components and the number of members from respective cadres of HCPs that should perform the identified workload component. The health service activities were not divided any further for which statistics were not available. Similarly, the participants were asked to reflect on activity standards. Activity standards were further bifurcated into service standards and allowance standards as per the methods highlighted in the manual. For support and additional activities, the participants were engaged in determining the allowance standard. The FGDs were transcribed, coded, and interpreted. For every cadre, subgroups, and specialties, health services activities, support activities, and additional activities were determined along with activity and allowance standards.

The validity of the FGD findings was assured by triangulation/cross-validation and respondent validation techniques [31, 32]. Nurses and doctors were observed in their respective settings by the researcher to verify the workload components and to check if some workload components whether any services activities, support activities, and additional activities were missed during FGDs. An extensive literature review of previously conducted WISN assessments in different settings was carried out before and after the FGDs to identify any missing workload components. The final report was presented to the HCPs of different cadres and specialties during a seminar to determine whether the participants felt the identified workload components and the allotted time were accurate. The opportunity was provided to them to comment on the finding, which could be later incorporated into the final calculations.

The tertiary healthcare hospitals of KP provide almost similar clinical services through their medical and allied specialties, surgical and allied specialties, and EDs. Therefore, health services activities and their activity standards had no significant difference. However, due to some differences in academic services related to undergraduate and postgraduate teachings and associated some clinical procedures, differences were observed in support and additional activities and their activity standards in three tertiary care hospitals, namely SGTH, LRH, and MTI Bannu. Based on the technical task force recommendations and agreement of the expert working group during the seminar, the health services activities and their activity standards were set similar for all the 8 tertiary care hospitals and support and additional activities and their activity standards were set individually for the hospitals due to the differences as shown in Tables 1, 2, 3, 4, 5 and 6.

**Table 1:** Health service activities and activity standards for doctors

Activity	Number of staff performing the work		Activity standard		Workload data	
*Medical and allied and surgical and allied specialties*						
Ward round	All doctors		7 min/patient		Inpatient days	
Admission	All doctors		15 min/patient		Number of admissions	
OPD	All doctors		10 min/patient		Number of OPD	
Death	All doctors		10 mints/death		Number of deaths	
Discharge	All doctors		10 min/patient		Number of discharges	
Pre- and post-round examination (DPR, order carryout, counseling, etc.)	All doctors		30 min/patient/day		Inpatient days	
Major surgeries*	All doctors		150 min/operation		OT data	
Minor surgeries*	All doctors		50 min/surgery		OT data	
*Emergency department*						
ED consultation	All doctors		10 min/patient		ED OPD	

*Specific for surgical and allied specialties

**Table 2:** Support activities and activity standards for doctors

Activity	Appropriate cadre	Activity standard
Post-graduate teaching (for all except MTI Bannu)	All doctors	180 min/week
Clinico-pathological conference	All doctors	60 min/week
Journal club	All doctors	60 min/week
Documentation	All doctors	60 min/week (For ED 180 min/wee)
Tea break	All doctors	30 min/day
Minor medical procedures (for medical and allied units only)	All doctors	180 min per week (for all hospitals except SGTH where it was 120 min/week)
Resuscitations and emergency patient care	All doctors	20 min/day (for ED 90 min/day)

**Table 3:** Additional activities and activity standards for doctors

Activity	Appropriate cadre		Activity standard
Undergrad teaching (except LRH)	Professorial staff		20 h /year
Committee/boards	Professorial staff		30 h/year
Performance appraisal	Professorial staff		2 h/year
Attending to call	2 doctors		60 min per day = 360 min per week
Administrative work (for ED only)	Shift in charge		3 h/day
Daily non-clinical round (for ED only)	In charge		6 h/week

**Table 4:** Health service activities and activity standards for nurses

Activity	Number of staff performing the work	Activity standard	Workload data
Medicine and allied and surgical and allied specialties			
Receiving, registering and admitting a patient	All nurses	15 min/patient	Admissions
Ward round	All nurses	7 min/patient	Inpatient days
Pre-ward and post-ward round orders (chart vital medication)	All nurses	30 min/patient	Inpatient days
Bedding	All nurses	5 min/patients	Inpatient days
Death care	All nurses	10 min/death	Number of deaths
Preoperative preparation*	All nurses	10 min/patient	OT data
Post-operative receiving/care*	All nurses	15 min/patient	OT data
Emergency department			
ED patient care	All nurses	5 min/patient	ED OPD

*Specific for surgical and allied specialties

**Table 5:** Support activities and activity standards for nurses

Activity	Appropriate cadre	Activity standard
Handing over taking over	All nurses	120 min/day (for ED 60 min/day)
Daily cleaning	All nurses	60 min/day
Detailed cleaning	All nurses	120 min/week
Ward meeting	All nurses	60 min/week
Tea break	All nurses	30 min/day
Assistance in medical procedures	All nurses	180 min per week (except SGTH where it was 60 min per week)

**Table 6:** Additional activities and activity standards for nurses

Activity	Appropriate cadre	Activity standard (medical and allied and surgical and allied)	Activity standard emergency department
Weekly medicine indent creation receiving and arranging	1 Charge nurse	120 min/week	60 min/week
Indent article daily	1 Charge nurse	60 min/day	60 min/day
Daily expanse	1 Charge nurse	60 min/day	60 min/day
Bedside teaching	1 Charge nurse	60 min/week	NA
Ward report	1 Charge nurse	90 min/day	NA
Administrative work	1 Charge nurse	60 min/day	30 min/day
Record keeping	1 Charge nurse	30 min/day	30 min/day
Resuscitation/emergency and on demand care	1 Charge nurse	60 min/week	180 min/day
Appraisals	Nursing superintendent	60 min/month	60 min/month
Duty roster	Nursing superintendent	120 min/month	NA

Annual service statistics of 2018 were collected from all the tertiary care hospitals of KP. Service statistics were collected from hospital HMIS for health services activities of both doctors and nurses working in medical and allied specialties, surgical and allied specialties, and ED. These collected statistics included department/specialty-wise and month-wise admissions, discharges, the daily number of patients in wards, OPD statistics (general and causality/ED), surgeries both major and minor, the average length of stay, bed occupancy, inpatient days among others. To ensure the accuracy of the data, the data generated from the HMIS system were cross-checked with individual ward’s daily statistics and OT and OPD records of every tertiary care hospital. The workload statistics and WISN calculations were recorded and analyzed using Microsoft Excel. The WISN software was not used for the final calculation due to challenges reported in the literature [33]. WISN was piloted with only one health staff cadre/category, i.e., for doctors in Ayub Teaching Hospital before the assessment was undertaken in all the tertiary care hospitals of the province. No results or estimates were generalized to other hospitals.

## Results

Staff requirements were calculated separately for doctors and nurses working in medical and allied specialties, surgical and allied specialties, and ED in all the eighth tertiary care hospitals of the province by initially estimating the annual workload of patients presenting to these hospitals and calculating their relative components as shown in Table 7

**Table 7:** Workload of the patients in the tertiary health care system

Hospital statistics	KTH		LRH	ATH		MMC		HMC		MTI DI Khan		MTI Bannu	SGTH
*Medical and allied specialties*													
Number of patients during ward rounds	150,956		194,767	172,339		150,100		165,537		160,520		101,119	210,369
Admission	35,106		51,535	44,324		38,886		43,911		57,534		36,114	61,332
OPD	332,911		445,788	412,944		175,622		290,058		131,582		281,844	220,701
Death	1653		6122	3621		2715		1885		2501		975	3139
Discharge	34,364		40,434	39,939		35,949		40,685		51,688		34,201	53,007
Number of patients during pre/post-round examination	150,956		194,767	172,339		150,100		165,537		160,520		101,119	210,369
*Surgical and allied specialties*													
Number of patients during ward rounds	186,180		239,496	188,702		165,916		193,133		95,154		112,221	229,433
Admission	40,474		88,876	51,455		36,707		41,996		24,336		33,006	52,382
OPD	336,471		511,385	276,690		216,150		277,357		124,713		192,361	285,022
Death	813		1888	489		97		1074		702		102	214
Discharge	38,294		69,646	49,867		34,952		39,699		21,441		31,892	45,676
Major surgeries	28,104		48,650	20,292		12,842		20,847		10,890		12,043	15,593
Minor surgeries	8267		60,017	30,168		8022		27,071		14,197		22,995	16,758
Number of patients during pre- and post-round examination	186,180		239,496	188,702		165,916		193,133		95,154		112,221	229,433
*ED visits*													
ED visits	673,998		1,189,663	323,964		226,601		590,091		212,650		248,770	409,835

The WISN results were analyzed by two methods. Firstly, the difference between the current and required number of staff was compared which indicated whether the hospital was understaffed or over staffed concerning specific cadre and specialty. WISN and its analysis were performed individually for each teaching hospital and the total health staff (doctors and nurses) needed in each teaching hospital was added to get the estimated health staff situation and work pressure in the tertiary care system of KP.

The results as shown in Table 8 indicate that individually all the hospitals showed a surplus of doctors except MTI DI Khan and MTI Bannu where there was a shortage of 55 and 181, respectively. However, the overall status of the tertiary healthcare system shows that there is a surplus of 1511 doctors in the tertiary health care system of the province.

**Table 8:** Healthcare staffing situation: doctors in tertiary healthcare system of KP

Health center	Doctors	Current number	Required number, based on WISN	Shortage or excess	Workforce problem	WISN ratio
Khyber Teaching Hospital	Medical and Allied	527	175	352	Surplus	3.01
	Surgical and Allied	537	249	288	Surplus	2.16
	Emergency	20	126	− 106	Shortage	0.16
	Total	1084	550	534	Surplus	1.97
Lady Reading Hospital	Medical and Allied	499	229	270	Surplus	2.18
	Surgical and Allied	536	411	125	Surplus	1.30
	Emergency	39	222	− 183	Shortage	0.18
	Total	1074	862	212	Surplus	1.25
Ayub Teaching Hospital	Medical and Allied	355	207	148	Surplus	1.71
	Surgical and Allied	351	244	107	Surplus	1.44
	Emergency	15	61	− 46	Shortage	0.25
	Total	721	512	209	Surplus	1.41
Mardan Medical Complex	Medical and Allied	178	147	31	Surplus	1.21
	Surgical and Allied	215	181	35	Surplus	1.19
	Emergency	18	43	− 25	Shortage	0.42
	Total	411	371	41	Surplus	1.11
Hayatabad Medical Complex	Medical and Allied	476	181	295	Surplus	2.63
	Surgical and Allied	518	242	276	Surplus	2.14
	Emergency	22	109	− 87	Shortage	0.20
	Total	1016	532	484	Surplus	1.91
MTI DI Khan	Medical and Allied	106	154	− 48	Shortage	0.69
	Surgical and Allied	138	121	17	Surplus	1.14
	Emergency	17	41	− 24	Shortage	0.41
	Total	261	316	− 55	Shortage	0.83
MTI Bannu	Medical and Allied	71	117	− 46	Shortage	0.61
	Surgical and Allied	52	154	− 102	Shortage	0.34
	Emergency	14	47	− 33	Shortage	0.30
	Total	137	318	− 181	Shortage	0.43
Saidu Teaching Hospital	Medical and Allied	408	195	213	Surplus	2.09
	Surgical and Allied	361	246	115	Surplus	1.47
	Emergency	16	77	− 61	Shortage	0.21
	Total	785	518	267	Surplus	1.52
Total across tertiary healthcare system		5490	3979	1511	Surplus	1.38

Specialty-wise, medical and allied and surgical and allied specialties showed a surplus of doctors in all the hospitals except MTI DI Khan, where there was a shortage of 48 doctors in medical and allied specialties, and MTI Bannu where every allied specialty showed a shortage of doctors.

Overall, there were 1215 surplus doctors in medical and allied specialties and 861 doctors in surgical and allied specialties in the tertiary healthcare system. However, the data analysis showed that the health care system had an acute shortage of 565 doctors in emergency departments. The same trend was observed in all the tertiary care hospitals of KP indicating an acute shortage of doctors in emergency departments.

In the second method of analysis, the WISN ratio was calculated by dividing the current number of staff by the required staff of specific cadre and specialty. This ratio is a proxy measure for work pressure the health worker will feel during his daily work. The lowest workload pressure based on the WISN ratio was observed in Khyber Teaching Hospital based on 2018 workload data where workload pressure was 1.92. Results show that the highest workload pressure was recorded in MTI Bannu where the WISN ratio was 0.43. For emergency departments of the tertiary hospitals, where every hospital’s data analysis showed acute shortage, the WISN ratio also indicated high workload pressure, and the highest workload pressure was recorded in the emergency department of KTH which was 0.16 followed by in LRH with 0.18 WISN ratio as shown in Table 8. The current number of doctors shown in Table 8 includes faculty of attached medical colleges, consultants, trainee registrars, medical officers, and residents.

Currently, 2517 nurses are working in the tertiary healthcare system of the KP province. The staffing assessment of nurses, as shown in Table 9, revealed that every tertiary care hospital in the province has an acute shortage of nurses. The highest shortage in terms of numbers was recorded in MMC with a shortage of 271 nurses based on the workload it received in 2018. LRH on the other hand had the highest number of nurses working among the tertiary care hospitals of the province and reported a shortage of only 29 nurses. The tertiary healthcare system of KP has an overall shortage of 1099 nurses which requires around 3616 nurses based on the workload of patients presenting to the tertiary healthcare system in 2018.

**Table 9:** Healthcare staffing situation: nurses in tertiary healthcare system of KP

Health center	Cadre	Current number	Required number, based on WISN	Shortage or excess	Workforce problem	WISN ratio
Khyber Teaching Hospital	Nurses	325	436	− 111	Shortage	0.74
Lady Reading Hospital	Nurses	773	802	− 29	Shortage	0.96
Ayub Teaching Hospital	Nurses	342	465	− 123	Shortage	0.73
Mardan Medical Complex	Nurses	109	380	− 271	Shortage	0.28
Hayatabad Medical Complex	Nurses	398	448	− 50	Shortage	0.88
Mufti Mehmood Teaching Hospital	Nurses	128	336	− 208	Shortage	0.38
Khalifa Gul Nawaz Teaching Hospital	Nurses	150	268	− 118	Shortage	0.55
Saidu Teaching Hospital	Nurses	292	391	− 99	Shortage	0.74
Total across tertiary healthcare system	Nurses	2517	3562	− 1099	Shortage	0.71

Further analysis of the departmental requirement of nurses was not carried out as the hospital data showed that the strength of various units changed during the year and there were very few specialized nurses working in the tertiary healthcare system and almost all of the nurses are general pool nurses which are rotated among various allied specialties and between specialties. Secondly, unlike doctors where the specialty matters for patient care, depending upon the public health emergency or disaster requiring a specific type of care, the principles of nursing care remain almost the same apart from exceptional circumstances.

Based on the WISN generated numbers for doctors, the estimated surge capacity of the tertiary health care system and its constituents tertiary care hospitals was calculated by the percentage difference between excess and required numbers of HCPs as highlighted in Tables 8 and 9. The HR surge capacity assessment as shown in Table 10 indicates that only four hospitals that are KTH, ATH, HMC, and SGTH have an adequate number of doctors to receive and manage a surge of ≥ 20% of patients in addition to their usual workload. However, the data show that if the surge capacity is estimated for individual specialties, none of the tertiary care hospitals’ EDs have any surge capacity in terms of the number of doctors to manage additional workload which is expected in the disaster scenarios. The tertiary healthcare system as a unit has the surge capacity to manage the additional patient workload of up to 27.5% with the current number of doctors. However, if the level of nursing care has to be maintained up to the locally acceptable standard of care and keeping in view the current workload, worryingly nor the tertiary healthcare system or any of its constituent tertiary care hospitals are prepared to manage any percentage of surge of additional of patients.

**Table 10:** Surge capacity of tertiary care hospitals

Health center	Cadre	Surge capacity %
Khyber Teaching Hospital	Medical and Allied	66.8
	Surgical and Allied	53.6
	Emergency Department	No surge capacity
	Total	49.3
Lady Reading Hospital	Medical and Allied	54.1
	Surgical and Allied	23.3
	Emergency	No surge capacity
	Total	19.7
Ayub Teaching Hospital	Medical and Allied	41.7
	Surgical and Allied	30.5
	Emergency	No surge capacity
	Total	29.0
Mardan Medical Complex	Medical and Allied	17.4
	Surgical and Allied	16.3
	Emergency	No surge capacity
	Total	10.0
Hayatabad Medical Complex	Medical and Allied	62.0
	Surgical and Allied	53.3
	Emergency	No surge capacity
	Total	47.6
MTI DI Khan	Medical and Allied	No surge capacity
	Surgical and Allied	12.3
	Emergency	No surge capacity
	Total	No surge capacity
MTI Bannu	Medical and Allied	No surge capacity
	Surgical and Allied	No surge capacity
	Emergency	No surge capacity
	Total	No surge capacity
Saidu Group Teaching Hospitals	Medical and Allied	52.2
	Surgical and Allied	31.9
	Emergency	No surge capacity
	Total	34.0

Nevertheless, the results show that if the total number of nurses and doctors are added and considered for joint surge response to a disaster, as shown in Table 11 and highlighted in the literature, only two hospitals in the province, i.e., KTH and HMC have adequate HCPs to manage a surge of ≥ 20% of patients. The provincial capital Peshawar has three tertiary care hospitals, i.e., KTH, LRH, and HMC. If the conglomerated surge capacity based on the combined number HCPs and their workload is considered in these three hospitals, the surge capacity is estimated to be 22.2% indicating that the provincial capital hospitals can jointly manage a sudden surge of patients and has the recommended surge capacity. The tertiary healthcare system of KP as a whole has a combined health workforce that can manage an additional surge of 6.3% of patients with the current patient workload.

**Table 11:** Surge capacity based on total numbers of health care providers

Hospital	Surge capacity (total healthcare providers)
KTH	30.0%
LRH	9.9%
ATH	8.1%
MMC	No (deficient HCP)
HMC	30.7%
MTI DI Khan	No (deficient HCP)
MTI Bannu	No (deficient HCP)
SGTH	15.6%
Conglomerated HMC, KTH, and LRH	22.2%
Tertiary healthcare system KP	6.3%

## Discussion

The phenomenon of the surge and its related capacity is not unidirectional or static. It has multiple components that intricately interact with each other to generate adequate surge capacity. The availability of appropriate numbers and a mix of HCPs are pivotal for the adequate surge capacity of the health system. This study was conducted to estimate the surge capacity of the tertiary healthcare system based on the actual workload of patients presenting to the tertiary care hospitals in the KP province.

Published literature on WISN seldom elaborates on the workload components and their activity standards and that makes it difficult to compare. Nevertheless, our workload components and activity standards were similar to what is reported in the literature. The results of our study show that the activity standard set for ward rounds was 7 min/patient which is similar to a study conducted in Uganda where ward round was set between 5 and 8 min [34]. Similarly, the activity standard for pre- and post-round patient care by nurses was set at 30 min which is similar to the routine nursing care activity standard (30 min) set for general hospitals in Namibia [33]. Available working days for doctors and nurses were recorded at 257 and 262 days, respectively, which far exceeds what has been reported in studies conducted in Asian and African hospitals that have reported between 199 and 246 available working days for various cadres of HCPs [34–37].

The study results show that individually all the hospitals have an excess of doctors except MTI DI Khan and MTI Bannu where there was a shortage of 55 and 181 doctors, respectively. However, the overall status of the tertiary health care system of the province shows that there is a surplus of 1511 doctors. This finding complements the results of the staffing need assessment conducted in Uganda which indicated that the clinical staff was adequate in their general hospitals [37]. However, very few studies have indicated an excess of doctors in hospitals [38]. Unlike the findings of our assessment, most of the studies have indicated a shortage of doctors in hospitals where WISN was conducted [33, 36, 39, 40].

Although WISN shows an overall surplus of doctors, yet all the tertiary care hospitals showed insufficient doctors in their EDs. The EDs everywhere are busy and the shortage of doctors and nurses based on the WISN calculations has been evident in other teaching hospitals as well. A study conducted in a teaching hospital in Iran showed a shortage and high work pressure of health care staff working in emergency departments [41]. Literature also suggests that emergency departments are usually understaffed whether assessed by WISN or otherwise [41–43]. In the context of Pakistan and KP particularly, these EDs work as evening OPDs and the patients usually present with minor ailment and complaints which causes an unnecessary workload in the EDs. This situation, however, is not exclusive to Pakistan or KP alone. Literature shows that even in advanced health systems like NHS in England, where there is a strong network of GPs, the patients still seek primary care in emergency departments of teaching hospitals/trust hospitals [44].

The results of this study show that all the tertiary care hospitals had an acute shortage of nurses with high workload pressure. Nurses more than any other cadre of health care providers are affected by the size, skills, and mix [34, 43]. Complementing the result of our survey, several studies both in developing and developed countries have reported an acute shortage of nurses working at every level of the healthcare delivery system [34, 36–39, 41–43, 45], Studies have shown that nurses have higher workload pressure as compared to doctors which indicates relatively fewer nurses as compared to doctors in the many healthcare delivery systems [36–38]. The result of this survey further strengthens these findings showing high workload pressure was more common in nurses as compared to doctors. The major reason for the shortage of nurses in the province is the lack of adequate nursing institutions and cultural barriers for the women to join this profession.

Several researchers have proposed role re-definition to overcome the shortage of healthcare providers during disasters where junior doctors or even medical and nursing students can provide necessary nursing care. Some studies have taken this interesting approach of role redefinition/re-designation towards filling the human resource gap and studied staffing assessment with combined healthcare staff. A study conducted in a large Turkish hospital that involved multiple cadres of staff, calculated the total staffing needs of the hospital and assessed the work pressure of the hospital instead of the individual cadre [36]. Sharing of roles between HCPs has been highlighted in other published literature as well, suggesting that the roles can be interchanged if a sudden surge in the number of patients is experienced [33]. Based on this approach, the results show that only three tertiary care hospitals, i.e., MMC, MTI DI Khan, and MTI Bannu have an overall shortage of healthcare workers. However, the tertiary healthcare system as a whole will still have surplus health care workers which can be deputed if the need arises to other hospitals as a measure of contingency and crisis surge capacity.

The criteria for surge capacity have been extensively discussed. It has been proposed by academics that the adequate level of surge capacity is the availability of 20% of total staffed beds for immediate management of patients during a public health emergency or disaster [13–15]. This number of 20% surge capacity in terms of availability of staffed beds is generated based on the US Health Resources and Services Administration advised capacity [14].

The current study shows that, based on the number of doctors, only four hospitals (KTH, ATH, HMC, and SGTH) can manage a sudden surge of ≥ 20% of patient workload and the tertiary healthcare system as a unit can manage an additional patient workload of up to 27.5%. Neither the tertiary healthcare system nor its constituent hospitals have a sufficient number of nurses that can manage any additional surge of patients.

However, for disaster response, if work pressure of the hospital instead of the individual cadre is taken as discussed earlier [36], only two hospitals in the province, i.e., KTH and HMC have adequate healthcare providers which may use role redefinition to manage a surge of ≥ 20% of patients if adequate beds and logistics are available. However, KP tertiary healthcare system’s HR as a whole can only manage an additional 6.3% of surge of patients. The results shows an insufficient surge capacity which stems from the shortage of doctors and nurses and is a major cause for concern for the health system decision-makers and hospital managers. Our study further augments the finding of the study conducted by Zia et al. that showed that the secondary health care system of KP province has poor preparedness for disaster management [46]. This highlights a critical fault in the overall health system of the province. In case of disaster, the tertiary healthcare system cannot rely on the secondary healthcare facilities to accommodate overflow surge of patients during disaster owing to the lack of preparedness and resources. The tertiary health care system and its constituent tertiary care hospitals have no option but to accommodate and manage whatever surge of patients they receive during disasters which will result in compromised quality of care. Having a sufficient HR surge capacity provides an opportunity and evidence for the health system to improve other surge capacity elements like beds, logistics, and finances to match HR capacity and achieve an adequate overall system surge capacity.

Some researchers argue that WISN uses previous year’s statistics to estimate staffing needs and it estimates staffing needs that should have been for the last year. However, the facility workload relatively changes slowly and the difference is usually non-significant from one year to another unless some special situation arises [23]. Thus, the estimate from the previous year may be true of the current year as well. As far as special situations, one can always apply percentage correction that can be factored in to calculate the estimate. As per the national disaster management authority and national counter-terrorism authority of Pakistan, KP experienced 2 natural and 310 terrorist activities during 2018. These disasters resulted in 275 deaths and 541 people presenting to various public sector hospitals with injuries. However, no disaster resulted in either a large influx of patients or a declaration of emergency by the hospitals or the provincial disaster management authority [47, 48]. Therefore, the estimates made using WISN fairly accurately represent the workload experienced during non-disaster situations in the tertiary healthcare system of KP.

## Conclusion

WISN assessment indicates that with the current workload, six out of eight tertiary care hospitals and the tertiary healthcare system as a unit have a surplus of doctors. However, the same cannot be said for nursing staff. None of the constituent hospitals of the tertiary healthcare system of KP have the required number of nurses to manage the current workload of patients.

Although, the conventional use of WISN assessment provides a robust workload based staffing situation and needs, yet, there is an untapped potential of the WISN technique to assess the staffing situation in certain scenarios where the hospitals might face a sudden surge of patients. WISN provides a novel approach to assess the surge capacity and resulting disaster preparedness of the tertiary healthcare system.

The HR surge capacity based on WISN indicates that the tertiary health care system of the Khyber Pakhtunkhwa Province of Pakistan does not possess the required ≥ 20% HR surge capacity indicating that the tertiary healthcare system is poorly prepared for disasters or public health emergencies even if other resources like beds and supplies are available. This lack of preparedness will compromise the quality and standards of care received by the disaster victims.

To improve the current HR situation and surge capacity, the tertiary healthcare system and its constituent hospitals need to hire and manage the current strength of HCPs by allocating them according to patient workload. To improve the surge capacity, it will not be cost effective to hire more HCPs preemptively to manage disasters. Instead, the health system must develop a provincial registry of nurses, volunteer and retired doctors, and allied staff that can be called if the need arises. Moreover, HCPs may be trained to adapt to role re-definition during disasters to manage the initial surge of patients before pre-identified HCPs from the pool can be deputed to the hospitals in need.

## Data Availability

All the relevant data and materials used in the study are available from author and can be provided upon reasonable request.

## Abbreviations

WHO: World Health Organization
HMIS: Health Management Information System
HCP: Healthcare Provider
HRH: Human Resources in Health
KP: Khyber Pakhtunkhwa
WISN: Workload Indicators of Staffing Needs
KTH: Khyber Teaching Hospital
LRH: Lady Reading Hospital
ATH: Ayub Teaching Hospital
HMC: Hayatabad Medical Complex
MTI DI Khan: Medical Teaching Institute Dera Ismail Khan
MMC: Mardan Medical Complex
MTI Bannu: Medical Teaching Institute Bannu
SGTH: Saidu Group of Teaching Hospitals
AWT: Available working time
ED: Emergency department
FGD: Focus group discussion
